# A Snapshot of the Trehalose Pathway During Seed Imbibition in *Medicago truncatula* Reveals Temporal- and Stress-Dependent Shifts in Gene Expression Patterns Associated With Metabolite Changes

**DOI:** 10.3389/fpls.2019.01590

**Published:** 2019-12-18

**Authors:** Anca Macovei, Andrea Pagano, Michela Cappuccio, Lucia Gallotti, Daniele Dondi, Susana De Sousa Araujo, Pedro Fevereiro, Alma Balestrazzi

**Affiliations:** ^1^Department of Biology and Biotechnology “L. Spallanzani,” University of Pavia, Pavia, Italy; ^2^Instituto de Tecnologia Química e Biológica António Xavier (ITQB-NOVA), Green-it Research Unit, Oeiras, Portugal; ^3^Department of Chemistry, University of Pavia, Pavia, Italy; ^4^Departamento de Biologia Vegetal, Faculdade de Ciências da Universidade de Lisboa, Lisboa, Portugal

**Keywords:** trehalose, seed imbibition, gene expression, abiotic stress, *Medicago truncatula*

## Abstract

Trehalose, a non-reducing disaccharide with multiple functions, among which source of energy and carbon, stress protectant, and signaling molecule, has been mainly studied in relation to plant development and response to stress. The trehalose pathway is conserved among different organisms and is composed of three enzymes: trehalose-6-phosphate synthase (TPS), which converts uridine diphosphate (UDP)-glucose and glucose-6-phosphate to trehalose-6-phosphate (T6P), trehalose-6-phosphatase (TPP), which dephosphorylates T6P to produce trehalose, and trehalase (TRE), responsible for trehalose catabolism. In plants, the trehalose pathway has been mostly studied in resurrection plants and the model plant *Arabidopsis thaliana*, where 11 *AtTPS*, 10 *AtTPP*, and 1 *AtTRE* genes are present. Here, we aim to investigate the involvement of the trehalose pathway in the early stages of seed germination (specifically, seed imbibition) using the model legume *Medicago truncatula* as a working system. Since not all the genes belonging to the trehalose pathway had been identified in *M. truncatula*, we first conducted an *in silico* analysis using the orthologous gene sequences from *A. thaliana*. Nine *MtTPS*s, eight *MtTPP*s, and a single *MtTRE* gene were hereby identified. Subsequently, the expression profiles of all the genes (together with the sucrose master-regulator *SnRK1*) were investigated during seed imbibition with water or stress agents (polyethylene glycol and sodium chloride). The reported data show a temporal distribution and preferential expression of specific *TPS* and *TPP* isoforms during seed imbibition with water. Moreover, it was possible to distinguish a small set of genes (*e.g.*, *MtTPS1*, *MtTPS7*, *MtTPS10*, *MtTPPA*, *MtTPPI*, *MtTRE*) having a potential impact as precocious hallmarks of the seed response to stress. When the trehalose levels were measured by high-performance liquid chromatography, a significant decrease was observed during seed imbibition, suggesting that trehalose may act as an energy source rather than osmoprotectant. This is the first report investigating the expression profiles of genes belonging to the trehalose pathway during seed imbibition, thus ascertaining their involvement in the pre-germinative metabolism and their potential as tools to improve seed germination efficiency.

## Introduction

Seed germination is a fundamental process for plant growth and development. It consists of a triphasic pattern determined by the dynamics of water uptake and reactivation of the seed metabolism. The first phase, representing the starting point of the pre-germinative metabolism, is seed imbibition. Imbibition is primarily a physical process defined by rapid water uptake, gas release, and temperature changes, taking place in both viable and nonviable seeds (Kranner et al., 2010; Rajjou et al., 2012). In viable seeds, the reactivation of dormant metabolism begins during phase I and continues in phase II, further characterized by active repair of membranes and DNA, initiation of protein synthesis, and mitochondrial activity, which drives the radicle emergence and entrance into the last phase of germination (Weitbrecht et al., 2011; Steinbrecher and Leubner-Metzger, 2017). The early phases of seed germination are essential for a robust seedling establishment, since they relate to key events that drive the seed from a quiescent state to the resurgence of embryo growth. Moreover, seed germination is conditioned by a wide variety of environmental stresses, ranging from water availability to soil conditions and temperature changes. To overcome stress, various mechanisms evolved to ensure a degree of resilience. To cite a few, the balanced reactive oxygen species (ROS) production/scavenging, DNA damage sensing and repair, or the accumulation of specific osmolytes (Ventura et al., 2012; Macovei et al., 2017; Farooq et al., 2017).

Within this context, sugars are important regulators of many physiological processes, including seed germination and stress response (Sami et al., 2016). For instance, high mannose and glucose concentrations had been demonstrated to inhibit *Arabidopsis* seed germination *via* the hexokinase signaling pathway (Pego et al., 1999). Often, these effects are correlated to phytohormone regulation. The ratio between sugar and hormone concentrations can either promote or inhibit germination (Gibson, 2004). Thus, aside energy-storing molecules, sugars are responsible for complex signaling and regulatory mechanisms, extensively studied in *Arabidopsis* sugar insensitive and hypersensitive mutants (Rolland et al., 2002). Recently, this dual function of sugars prompted the use of integrative metabolomics analyses to investigate their complex roles during seed germination in several plant species (Kazmi et al., 2017; Silva et al., 2017; Biswas et al., 2018; Chen et al., 2019).

Among the different types of sugars, trehalose, a non-reducing disaccharide composed of two D-glucose molecules linked with a α,α-1,1-glycosidic bond, is known to have multiple functions, like source of energy and carbon, stress protectant, and signaling molecule (Elbein et al., 2003; Paul et al., 2008; Iturriaga et al., 2009; Delorge et al., 2014). Trehalose has been studied in many model organisms, from *Escherichia coli* and *Saccharomyces cerevisiae* to *Drosophila melanogaster* and *Arabidopsis thaliana*, and the components of its metabolic pathway are structurally and functionally conserved (Strøm and Kaasen, 1993; Hounsa et al., 1998; Avonce et al., 2006; Schluepmann and Paul, 2009). Namely, the trehalose pathway is composed of three enzymes: 1**)** trehalose-6-phosphate synthase (TPS), which converts uridine diphosphate (UDP)-glucose (UDP-Gluc), and glucose-6-phosphate (G6P) to trehalose-6-phosphate (T6P); 2**)** trehalose-6-phosphatase (TPP), which dephosphorylates T6P to produce trehalose; and 3**)** trehalase (TRE), responsible for trehalose catabolism, splitting it into two glucose molecules. The T6P metabolic intermediate is regarded as a key component of regulatory mechanisms controlling plant growth and development, mainly through the regulation of carbohydrate metabolism and allocation (Ponnu et al., 2011; Figueroa and Lunn, 2016). This is thought to be achieved by the direct links existing between T6P and the SnRK1 (sucrose-non-fermenting1-related kinase) family, acting as sensors of the energy level (Tsai and Gazzarrini, 2012; Figueroa and Lunn, 2016).

In plants, the genes involved in the trehalose biosynthetic pathway have been first described in *A. thaliana* (Blazquez et al., 1998; Vogel et al., 1998) and subsequently identified, with different degrees of homology, in other commercially-relevant species, such as rice, maize, potato, tobacco, canola, and cotton (Lunn et al., 2014). In *A. thaliana*, 11 *TPS* genes (*AtTPS1-11*), 10 *TPP* genes (*AtTPPA-J*), and a single *TRE* gene (*AtTRE*) have been identified (Leyman et al., 2001; Schluepmann et al., 2004; Avonce et al., 2006). The AtTPS enzymes have been divided into two classes in relation to the presence (class I, AtTPS1-4) or absence (class II, AtTPS5-11) of the conserved glucosyltransferase-like domain. The functional landscape of TPS classes is variegated, since the class I AtTPS enzymes possess catalytic activity able to complement yeast mutants, whereas class II AtTPS proteins do not (Ramon et al., 2009; Vandesteene et al., 2010). Nonetheless, it has been argued that class II AtTPS proteins may have regulatory rather than metabolic functions since their expression appears to be tissue-specific and regulated in response to growth hormones and carbon availability (Ramon et al., 2009). Conversely, heterologous expression of *AtTPP* genes in *S. cerevisiae* has shown that all their encoded proteins are catalytically active. Their expression patterns are also influenced by developmental stage and tissue specificity (Paul et al., 2008; Vandesteene et al., 2012).

Even though the trehalose pathway has been extensively studied in the *Arabidopsis* model, mostly in connection to plant growth and development, its relation to the seed germination process is still largely overlooked. A recent study reported the accumulation of trehalose in response to seed priming applied to overcome low-temperature stress in chickpea (Farooq et al., 2017). This resulted in improved germination since the seeds had better carbon assimilation and protection against oxidative damage. However, this study focused more on the physiological and biochemical parameters of seedling growth, still leaving a gap of knowledge concerning the molecular aspects, especially related to the early phases of seed germination, *e.g.*, seed imbibition. To fill-in this existing gap, the present study aimed to look into the molecular aspects of the trehalose pathway during seed imbibition in the model legume *Medicago truncatula*. This species plays an important agronomic role as forage crop in dryland farming and as an integral component of intercropping systems, particularly in US, Australia, and Mediterranean regions (Piano and Francis, 1992; Sheaffer and Lake, 1997; Walsh et al., 2001), but its yield is severely affected by water limitation, especially during seed germination (Carrillo-Reche et al., 2018). Because not all the genes belonging to the trehalose pathway were identified in *M. truncatula*, we first proceeded with a database mining to complete this pathway and then investigated the expression profiles of these genes during seed imbibition under physiological and stress conditions induced by polyethylene glycol (PEG) and sodium chloride (NaCl). Subsequently, the trehalose levels were measured under the same conditions. The results presented here evidence temporal- and stress-dependent shifts in gene expression patterns associated with metabolite changes. This suggests that specific *TPS* or *TPP* genes could be used as indicators of seed vigor or stress responses during the early phases of seed germination.

## Materials and Methods

### Bioinformatic Analyses

*A. thaliana* gene sequences encoding enzymes from the trehalose pathway were used to identify *M. truncatula* orthologues through BLAST tool available within the Phytozome database (https://phytozome.jgi.doe.gov/pz/portal.html) (Goodstein et al., 2012). The protein sequences were compared using Clustal Omega alignment tool (https://www.ebi.ac.uk/Tools/msa/clustalo/), to determine the similarity percentage. Only the *M. truncatula* proteins with a similarity ≥ 50% with their *A. thaliana* orthologs were considered for further analysis. The architecture of the conserved domains of *M. truncatula* proteins was recovered through the Simple Modular Architecture Research Tool (http://smart.embl.de/) tool (Letunic et al., 2015) and graphically represented using the IBS (Illustrator for Biological Sequence) (http://ibs.biocuckoo.org/) software (Liu et al., 2015). Only the proteins with complete TPP or TRE domains were retained. For this selection step, the Conserved Domain Search of NCBI database (https://www.ncbi.nlm.nih.gov/Structure/cdd/wrpsb.cgi) was used. The BAR (Bio-Analytic Resource) tool (http://www.bar.utoronto.ca/) was used to retrieve the expression profiles of genes from the trehalose pathway during seed germination in *A. thaliana* (Winter et al., 2007; Bassel et al., 2008).

### Plant Material and Treatments

*M. truncatula* seeds (Jemalong commercial cultivar, kindly provided by Fertiprado L.d.a., Vaiamonte-Monforte, Portugal) were used in this study. To test the germination potential, seeds were transferred to Petri dishes (90 mm) containing two filter papers moistened with 2 mL distilled H_2_O (CTRL) and monitored for 14 days. Germination was also evaluated in response to different osmotic stresses stress treatments applied by using polyethylene glycol (PEG6000) and sodium chloride (NaCl). For this, seeds were imbibed in presence of PEG6000 (Sigma-Aldrich, Milan, Italy), with concentrations of 50 g L^-1^ and 100 g L^-1^ (hereby referred to as PEG50 and PEG100 samples, respectively), and NaCl (Duchefa Biochemie, Haarlem, The Netherlands) with concentrations of 50 mM and 100 mM (hereby referred to as NaCl50 and NaCl100 samples, respectively). The Petri dishes were sealed with paraffine films and kept in a growth chamber at 22°C under light conditions with a photon flux density of 150 µmol m^-2^ s^-1^, photoperiod of 16/8 h and 70-80% relative humidity (RH).

### Measurement of Germination Parameters

Treated and untreated seeds were imbibed and germinated in parallel, under the aforementioned conditions. Seed weight was measured every two hours (0, 2, 4, 6, and 8 h) during imbibition to estimate water uptake. Seeds with protrusions of the primary radicle (>1 mm) were considered germinated. Germinated seeds were checked daily and germination was followed for 14 days. Imbibition and germination data were collected from two independent experiments carried out with three replicates (each containing 20 seeds) for each treatment. Germination percentage was calculated daily as the total number of germinated seeds on the total number of sown seeds and used as an indicator of seed viability. The T_50_ values were calculated according to Farooq et al. (2005) as the time necessary to reach 50% of germinated seeds and used as a parameter to describe germination speed.

### Reactive Oxygen Species Detection by 2’,7’-Dichlorofluorescein Diacetate Staining

2’,7’-Dichlorofluorescein diacetate (DCFA-DA) is a fluorogenic dye used to detect the presence of ROS within a sample. After diffusion into the cell, DCFA-DA is deacetylated by cellular esterase enzymes to a non-fluorescent compound, which is later oxidized by ROS into 2’,7’-dichlorofluorescein (DCF), thus allowing ROS detection. DCF is a highly fluorescent compound which can be detected by fluorescence spectroscopy with maximum excitation and emission spectra between 495 and 529 nm (Rastogi et al, 2010). Treated (PEG100 and NaCl100) and untreated (CTRL) *M. truncatula* seeds were collected at the indicated time points (0, 2, 4, 6, and 8 h). After imbibition, the seeds were dried with filter paper and used for DCFA-DA staining as previously described (Macovei et al., 2016). A stock solution of 10 mM solution DCFA-DA (Sigma Aldrich, Italy) was diluted to 10 µM before use. The seeds (three seeds per treatment/timepoint) were incubated for 15 min with 50 µl of 10 µM DCFH-DA under dark conditions. Relative fluorescence was determined at a wavelength of 517 nm in a Rotor-Gene 6000 PCR apparatus (Corbett Robotics, Brisbane, Australia), setting the program for one cycle of 30 s at 25°C. A negative control containing only DCFA-DA was used to subtract the baseline fluorescence, thus calculating the relative fluorescence (represented as RFU, relative fluorescence units) for each sample.

### Nontargeted Metabolomic Profiling

To quantify sugar metabolites, a nontargeted metabolomic profiling was performed by Metabolon Inc. (Durham, NC, U.S.A.; www.metabolon.com), based on three independent platforms: ultrahigh-performance liquid chromatography/tandem mass spectrometry (UHLC/MS/MS2) optimized for basic species, UHLC/MS/MS2 optimized for acidic species, and gas chromatography/mass spectrometry (GC/MS). Global metabolomic profiling was performed on methanol extracts from powdered frozen samples (100 mg) of dry seeds (DS) and seeds imbibed with H_2_O for 2 and 8 h. Metabolomic analyses were conducted in biological triplicates, each containing at least 20 seeds. Metabolites were identified by automated comparison of the ion features of the experimental samples with a reference library of chemical standard entries that included retention time, molecular weight (m/z), preferred adducts, and in-source fragments as well as associated MS/MS2 spectra and curated by visual inspection for quality control using in-house resources (Evans et al., 2009; Oliver et al., 2011). Raw area counts for each biochemical compound were normalized by dividing each sample value per sample fresh weight. Before data analysis, scaled imputed data were log-transformed. Metabolites that achieved statistical significance of p ≤ 0.05 and q ≤ 0.1 were considered. For metabolomic profiling analysis, Welch’s two sample *t*-tests were used to determine whether each metabolite had significant changes in abundance, focusing on comparisons between DS and imbibed samples. Heatmap and clustering analysis were conducted using the software resources available at MetaboAnalyst 4.0 (www.metaboanalyst.ca) (Chong and Xia, 2018).

### Detection of Trehalose Content

To prepare the standards and samples, 1 g of treated/untreated seeds were transferred in a Teflon tube and suspended by sonication in 2 ml of eluent mixture MeCN:H_2_O (80:20). The same used as mobile phase for high-performance liquid chromatography (HPLC) analysis. Each sample solution was filtered through a nylon filter (0.45 µm pore size) before injection. The standard solution was represented by 2 ml eluent mixture solution containing 1 g of trehalose (Sigma-Aldrich, Milan, Italy) powder. HPLC chromatograms were recorded with a Waters 1525 Binary HPLC Pump (^©^2008 Waters Corporation) equipped with a Waters 2414 Refractive Index Detector system (^©^2008 Waters Corporation) having a light-emitting diode (LED) light source, fused quartz flow cell, volume 10 µl, pressure limit 100 psi. The system is thermostated at 40°C and the injection loop volume was 25 µl. A LunaNH2^®^ (Phenomenex, 5 µm, 250x4.6 mm) column, including guard column, was used to optimize the retention of simple sugars (increased bonded phase stability, pH stability from 1.5 up to 11). The eluent mixture used as mobile phase (80:20 acetonitrile/water v/v) was filtered through a nylon filter (0.45 µm pore size) and degassed with ultrasonic bath for preventing out-gassing problems and interferences. The flow rate was 3 ml/min. All injections were carried out for a run time of 15 min to better ensure the total elution of the sample. The peaks of HPLC spectra were integrated and compared to the trehalose standard signal previously acquired. The concentration values were obtained on an average of three injections for each sample. The calibration curve was obtained for a concentration of trehalose in the range 3x10^−2^ − 1.5x10^−4^ M (R^2^ = 0.998).

### Ribonucleic Acid extraction, Complementary Deoxyribonucleic Acid Synthesis, and Quantitative Real-Time Polymerase Chain Reaction

RNA isolation was carried out as described by Oñate-Sanchez and Vicente-Carbajosa (2008). RNA was extracted from three biological replicates consisting of a pool of seeds (approximately 100 mg each). To remove DNA, RNA samples were treated with DNase I, RNase-free (1U µL-1) (ThermoFisher Scientific, Milan, Italy), according to manufacturer’s suggestions. cDNAs were obtained using the RevertAid First Strand cDNA Synthesis Kit (ThermoFisher Scientific) according to the manufacturer’s suggestions. Quantitative real-time polymerase chain reaction (qRT-PCR) was performed with the Maxima SYBR Green qPCR Master Mix (2X) (ThermoFisher Scientific) according to supplier’s indications, using a Rotor-Gene 6000 PCR apparatus (Corbett Robotics Pty Ltd., Brisbane, Queensland Australia). Amplification conditions were as follows: denaturation at 95°C for 10 min, 45 cycles of 95°C for 15 s and 60°C for 60 s. The primers, listed in **Supplementary Table 1**, were designed using the Primer3Plus (http://www.bioinformatics.nl/primer3plus) software (Untergasser et al., 2007) and further verified with OligoAnalyzer (https://eu.idtdna.com/calc/analyzer). The following genes were analysed: MtTPS1 (Medtr2g073260), MtTPS2 (Medtr8g087930), MtTPS5 (Medtr1g109620), MtTPS6 (Medtr8g105740), MtTPS7 (Medtr4g080160), MtTPS8 (Medtr3g078210), MtTPS9 (Medtr8g063790), MtTPS10 (Medtr1g032730), MtTPS11 (Medtr4g12927), MtTPPA (Medtr4g036090), MtTPPB (Medtr3g074180), MtTPPC (Medtr5g063080), MtTPPE (Medtr8g027765), MtTPPF (Medtr3g008500), MtTPPG (Medtr4g036685), MtTPPH (Medtr4g101600), MtTPPI (Medtr8g090330), MtTRE (Medtr8g099985), and MtSnRK1 (Medtr6g012990). Quantification was carried out using actin (MtACT, Medtr3g095530) as a reference gene. Initially, both actin and tubulin genes were considered, but since the tubulin gene expression proved to be unstable during the tested conditions, only the actin gene was maintained as stable reference gene. The raw, background-subtracted fluorescence data provided by the Rotor-Gene 6000 Series Software 1.7 (Corbett Robotics) was used to estimate PCR efficiency (E) and threshold cycle number (Ct) for each transcript quantification and the Pfaffl method (Pfaffl, 2001) was used for relative quantification of transcript accumulation. The expression profiles relative to stress treatments are represented as fold-change (FC) to respective controls (imbibition in water). The data is represented as heatmap constructed using the ShinyHeatmap application available at http://www.shinyheatmap.com/ (Khomtchouk et al., 2017).

### Statistical Analysis

For each analysis, three biological replicates were considered. Statistical analysis was performed using the two-way ANOVA *post hoc* pairwise comparisons (PHC) and Tukey-Kramer (Tukey’s HSD) test (Assaad et al., 2015). Significant differences among samples are represented with lowercase letters. The principal component analysis (PCA) was performed using the ClustVis software, a web tool for the visualization and clustering of multivariate data (BETA), available at https://biit.cs.ut.ee/clustvis/ (Metsalu and Vilo, 2015). Pattern search analysis was conducted using the PatternHunter tool (relying on Pearson correlation coefficient) available at https://www.metaboanalyst.ca/MetaboAnalyst/faces/Secure/analysis/PatternView.xhtml (Chong and Xia, 2018). Correlation analysis was performed against “trehalose” as a given feature. The pattern is specified as a series of numbers separated by “-.” Each number corresponds to the expected expression pattern in the corresponding group. The order of the groups is given as the first item in the predefined patterns.

## Results

### Bioinformatic Reconstruction of the Components Belonging to the Trehalose Pathway in *Medicago truncatula*

**Figure 1:**
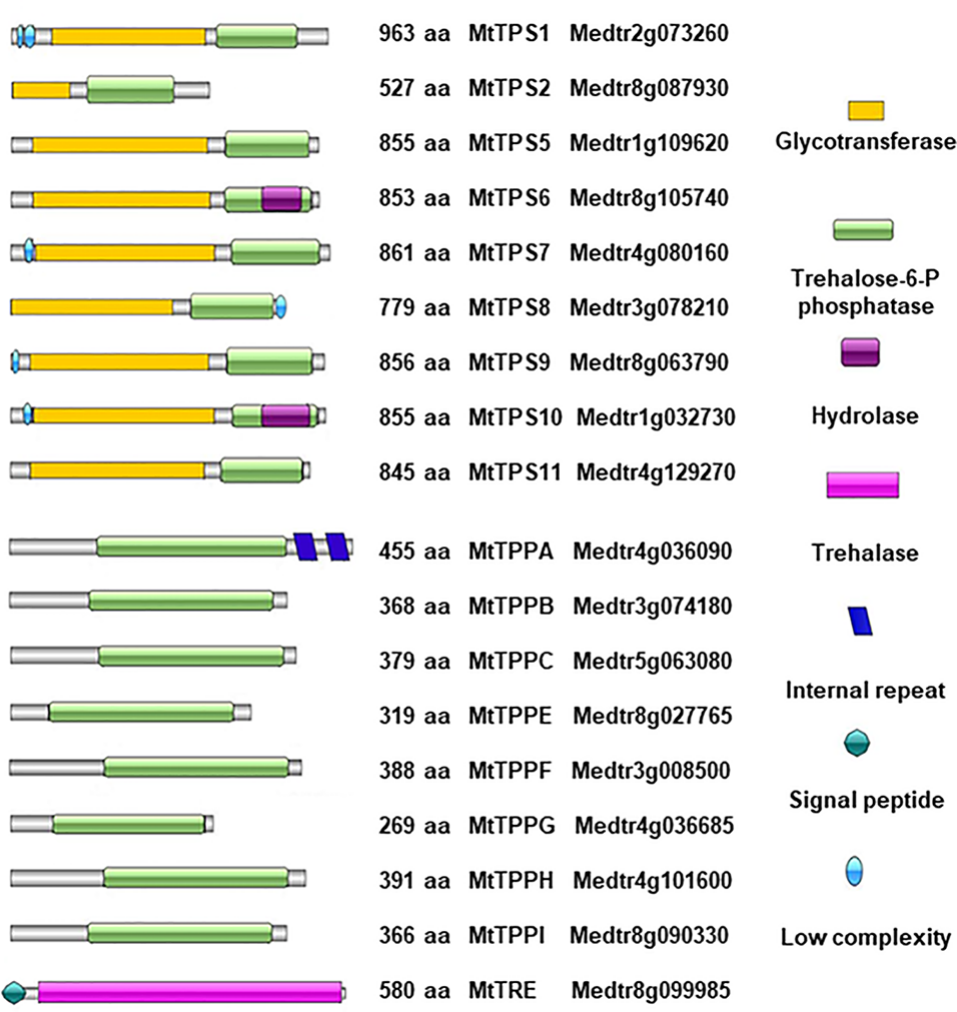
Domain architecture of *Medicago truncatula* MtTPS, MtTPP, and MtTRE proteins. Domain names are given according to SMART (Simple Modular Architecture Research Tool). Protein length and Phytozome accession numbers are indicated for each isoform; aa, amino acid.

To retrieve the genes belonging to the trehalose pathway in *M. truncatula*, the *A. thaliana* sequences were blasted against the *M. truncatula* genome using the tools available in the Phytozome database. The accession numbers of the genes encoding the enzymes of the trehalose pathway, in both species, are presented in the **Supplementary Table 2**. It is possible to observe that, aside from the *TPS1* and *TRE* where only one ortholog was identified also in *M. truncatula*, the rest of the genes gave multiple hits, with *TPS5* (eight hits) and *TPPF* (nine hits) presenting the highest number of accessions. However, most of the orthologues overlap between the different categories of genes. To better distinguish among them, the *A. thaliana* and *M. truncatula* protein sequences were aligned to determine their percentage of similarity and only *M. truncatula* proteins with a similarity ≥ 50% with their *A. thaliana* orthologs were retained for further bioinformatics analysis. A second threshold was imposed based on protein sequence integrity, thus eliminating all orthologs that presented truncated domains. The architecture of the conserved domains was reconstructed and graphically represented in **Figure 1**, where only the proteins with complete TPP domains or complete TRE domains are listed. These include nine MtTPS proteins, eight MtTPP proteins, and a single MtTRE protein. The length of each protein is annotated along with its domain architecture and the accession number of the gene encoding it. Among the class I TPS, *M. truncatula* seems to have retained only the functional TPS1 (Medtr2g073260) and a shorter putative TPS2 (Medtr8g087930) protein, as the orthologues relative to TPS3 and TPS4 showed truncated protein domains or were located on pseudochromosomes (**Supplementary Table 2**). A similar situation was encountered in the case of TPPD and TPPJ proteins. The MtTPS isoforms contain functional glycosyltransferase domains at the N-terminus and T-6-P phosphatase domains at the C-terminus. A hydrolase domain is overlapping with the T-6-P phosphatase domain in two MtTPS isoforms (MtTPS6-Medtr8g105740 and MtTPS10-Medtr1g032730). Low complexity regions have been identified at the N-terminus of four MtTPS (MtTPS1, MtTPS7, MtTPS9, MtTPS10) proteins and at the C-terminus of one MtTPS (MtTPS8) protein. A T-6-P phosphatase domain is present in all the listed MtTPP isoforms. The TRE domain found in the MtTRE protein is also shown.

### Seed Imbibition Triggers Changes in the Expression of Genes Encoding Enzymes From the Trehalose Pathway Along With Alteration of the Carbohydrate Metabolism in *Medicago truncatula*

**Figure 2:**
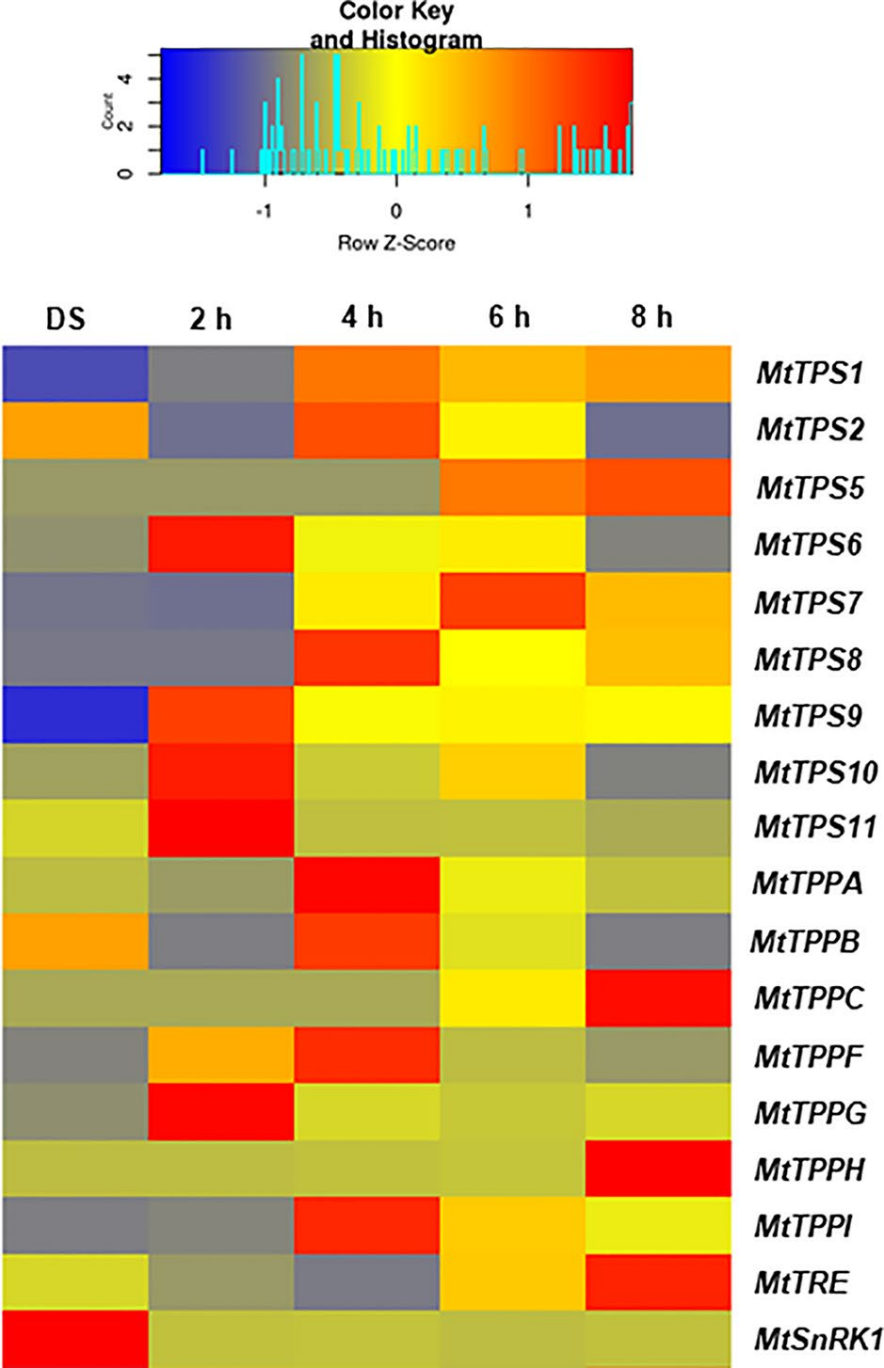
Heatmap representing the expression profiles of *Medicago truncatula MtTPS*, *MtTPP*, *MtTRE*, and *MtSnRK1* genes in dry seed (DS) and during seed imbibition with water at the indicated timepoints (2, 4, 6, and 8 h, respectively). Expression levels are represented by with color codes, where red is the highest expression while blue is the lowest expression.

Following the bioinformatic identification of *M. truncatula TPS*, *TPP*, and *TRE* genes, qRT-PCR was carried out to investigate gene expression during regular seed imbibition (**Figure 2**). The expression of most genes is quite low in DS. Among the *MtTPS* genes, *MtTPS6*, *MtTPS9*, *MtTPS10*, and *MtTPS11* were upregulated already after 2 h of imbibition. At 4 h of imbibition, *MtTPS1*, *MtTPS2*, and *MtTPS8* were the most expressed whereas, after 6 h, *MtTPS5*, and *MtTPS7* showed the highest expression level. Finally, at 8 h, the most expressed gene was *MtTPS5*. The *MtTPS1* expression was maintained at elevated levels also at 6 and 8 h of imbibition. It is interesting to note the fact that while *MtTPS6*, *MtTPS9*, *MtTPS10*, and *MtTPS11* are highly expressed at 2 h of imbibition, at the subsequent timepoints their expression declines considerably. When considering the *MtTPP* genes, *MtTPPG* is most expressed at 2 h, *MtTPPA*, *MtTPPB*, *MtTPPF*, and *MtTPPI* are most expressed at 4 h, while *MtTPPC* and *MtTPPH* are most expressed at 8 h of imbibition. As for the *MtTRE* gene, the highest expression was registered at 8 h of imbibition. Overall, these data show a temporal distribution and preferential expression of specific *TPS* and *TPP* isoforms during seed imbibition. Moreover, *A. thaliana* microarray data supports for a similar conclusion (**Supplementary Figure 1**). Although the corresponding time points of imbibition and upregulated isoforms does not completely match, it is at least confirmed that *TPS6* and *TPS11* have the earliest upregulation response in both species (1 h for *A. thaliana* and 2 h for *M. truncatula*) (**Supplementary Figure 1**).

Because of the roles that snrk1 have in regulating sugar metabolism, its expression levels were also evaluated in this study. The expression profiles of *MtSnRK1* show that the gene is highly expressed in ds and its expression subsequently decrease during seed imbibition (**Figure 2**). This is in agreement with the fact that the SnRK1 is known to regulate seed maturation and obstructs germination in relation to the abscisic acid signaling pathway (Radchuk et al., 2010).

**Figure 3:**
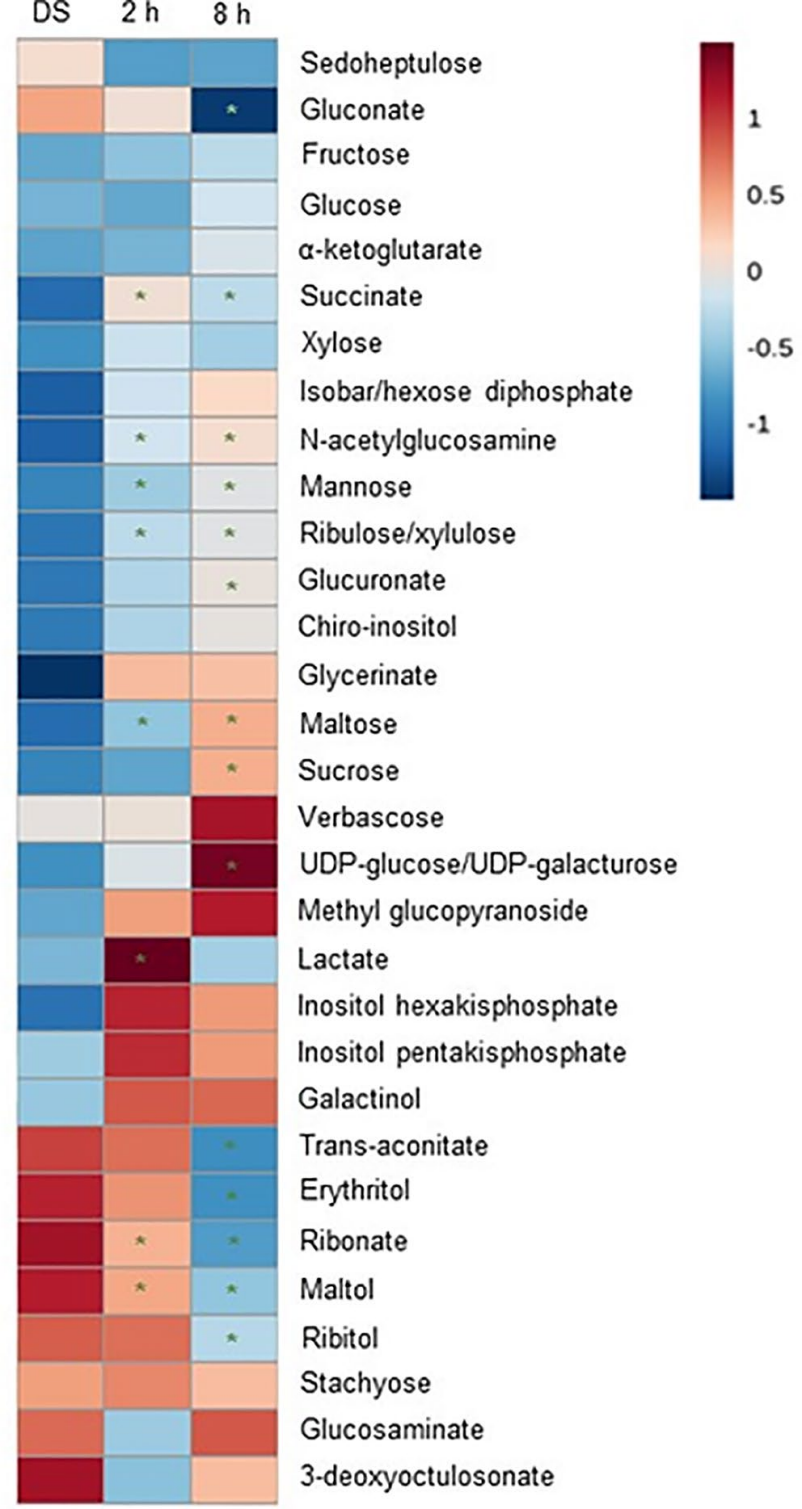
Heatmap of the 31 metabolites belonging to the carbohydrate metabolic pathway with significant changes (p ≤.05 and q ≤.1) during *Medicago truncatula* seed imbibition (at 2 and 8 h, respectively). The color (from red to blue) represents the mean value log transformed raw-scaled imputed value for each metabolite from high to low, respectively. Statistical significance (“*”) is given by Welch’s two-sample *t*-tests, focusing on comparisons between dry seeds (DS) and imbibed samples.

As different metabolic pathways are being reactivated during seed imbibition, in previous works we monitored the changes in metabolite profiles using a non-targeted metabolomics approach (Pagano et al., 2019; Araújo et al., 2019). From the generated datasets, here we have extrapolated only the changes in the carbohydrate metabolic pathway since trehalose is included in this category (**Figure 3**). Among the 31 metabolites associated with the carbohydrate metabolic pathway, the levels of succinate, mannose, ribulose, and maltose were significantly enhanced while the levels of ribonate and maltol were significantly decreased at both tested time points (2 and 8 h) of imbibition as compared to DS. Other significant (*P* < 0.05) changes in the carbohydrate levels during the transition from the DS to imbibed seeds were registered only at 8 h of imbibition when decreased levels of gluconate, trans-aconitate, erythritol, and stachyose were observed along with increased levels of glucuronate, sucrose, and UDP-glucose/UDP-galacturose, while the lactate levels were enhanced only at 2 h of imbibition. Although trehalose was not detected with this approach, the increase in UDP-glucose at 8 h of imbibition may indicate subsequent load into the trehalose metabolic pathway, since it represents one of the main starting substrates for this pathway. Moreover, the increased sucrose levels could be correlated with the observed decrease in the *MtSnRK1* gene expression, as previously reported in *A. thaliana* seedlings (Williams et al., 2014).

### Osmotic and Salinity Stresses Affect Seed Germination and Induce Reactive Oxygen Species Accumulation During the First Hours of Imbibition

Because trehalose is supposed to be involved in the plant response to abiotic stresses (Iturriaga et al., 2009; Delorge et al., 2014), we decided to carry out imbibition with stress agents (PEG, NaCl). Initially, two concentrations of PEG (50 and 100 g L^−1^) and NaCl (50 and 100 mM) were tested to evaluate their influence on seed germination (**Supplementary Figure 2**). During seed imbibition, water uptake was significantly reduced only when PEG100 was used (**Supplementary Figures 2A, B**). On the other hand, a significant decline in seed germination percentage was observed in the presence of both PEG100 and NaCl100 treatments (**Supplementary Figure 2C**). Similarly, the germination speed has changed, showing that PEG100- and NaCl100-treated seeds necessitate 5 to 7 days to reach T_50_ whereas water-imbibed seeds achieved it in 2 days (**Supplementary Figure 2D**). Based on these preliminary analyses, the treatments that resulted in significant differences (PEG100 and NaCl100, respectively) compared to untreated control (water) were selected for further characterization.

**Figure 4:**
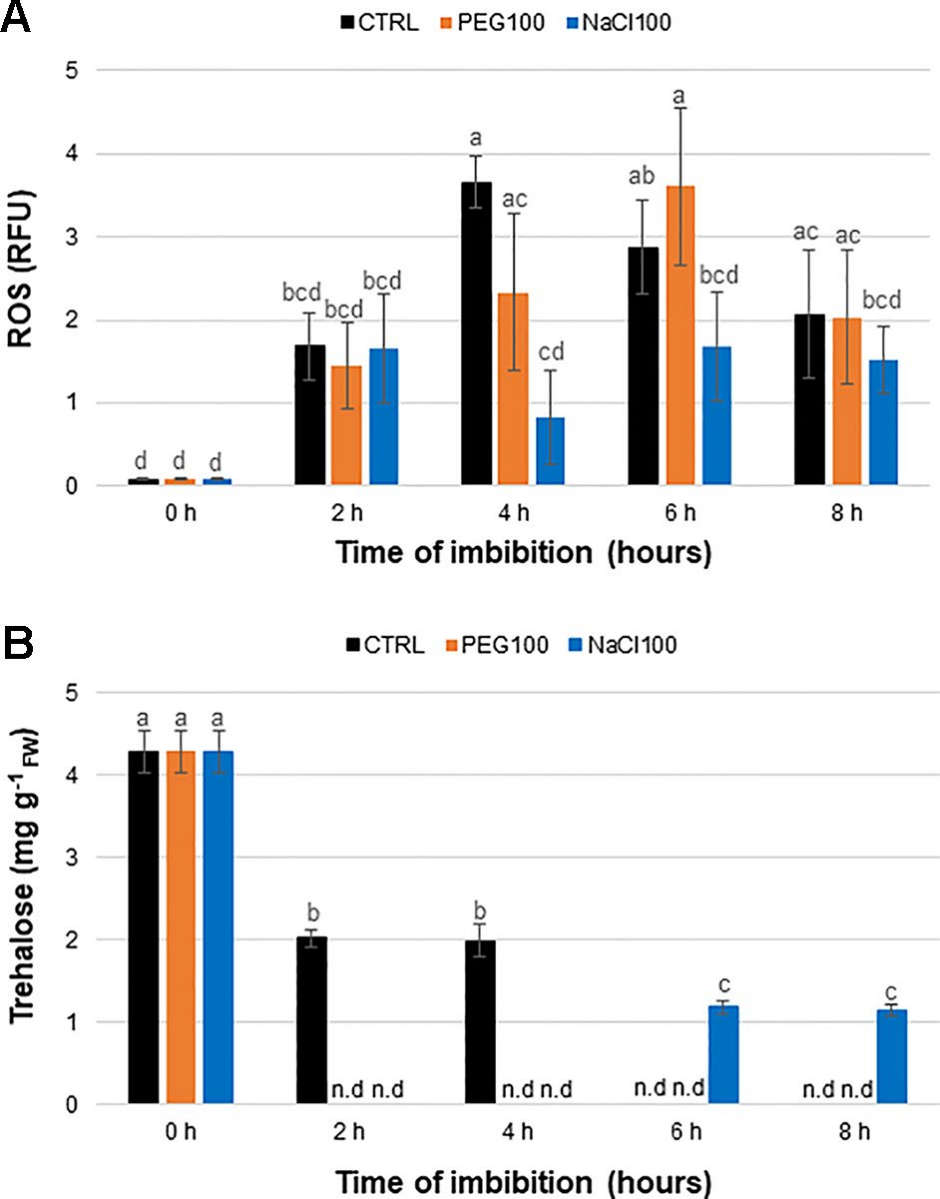
Detection of reactive oxygen species **(A)** and trehalose levels **(B)** in *Medicago truncatula* seeds during imbibition with water (CTRL), polyethylene glycol 100 g L^−1^ (PEG100), and sodium chloride 100 mM (NaCl100). Significant differences (as per Tukey’s test) are shown with lowercase letters; n.d., not determined.

The ROS levels were minimal in DS (0 h) while the highest peak was registered at 4 h of imbibition with water and slowly decreased at the subsequent time points (**Figure 4A**). In the case of PEG100 treatment, the maximum peak was observed at 6 h of imbibition, in agreement with the slower water uptake observed in this case. As for the NaCl100 treatments, although fluctuations in the ROS levels are observed, these are less prominent than those observed during imbibition with water or PEG.

### Trehalose Content Decreases During Seed Imbibition as Revealed By High-Performance Liquid Chromatography Analyses

HPLC analyses were conducted on water-, PEG100-, and NaCl100-imbibed seeds throughout the time course of the experiment (0, 2, 4, 6, and 8 h). The peaks of HPLC spectra were integrated and compared to the trehalose standard and the concentration is represented in mg g^−1^ (**Figure 4B**). The calibration curve, obtained for a concentration of trehalose in the range 3x10^−2^ – 1.5x10^−4^ M (R^2^ = 0.998), is shown in **Supplementary Figure 3**. Our results show that the highest trehalose content was detected in DS (4.29 ± 0.25 mg g^−1^, 0 h), and subsequently it decreased during imbibition with water at 2 h (2.02 ± 0.1 mg g^−1^) and 4 h (1.99 ± 0.19 mg g^−1^) while trehalose was no longer detected at 6 and 8 h (**Figure 4B**, CTRL). During imbibition with PEG100, trehalose was non-detectable (**Figure 4B**, PEG100, n.d), possibly because PEG showed a reduced sensitivity and a poor linearity (R^2^ = 0.744) with respect to curves made at the same concentration in water and NaCl (data not shown). Trehalose was detected during imbibition with NaCl100 only after 6 h (1.18 ± 0.08 mg g^−1^) and 8 h (1.13 ± 0.07 mg g^−1^), at significantly lower concentrations when compared to DS (0 h) (**Figure 4B**, NaCl100). It is interesting to note the fact that, while during imbibition with water trehalose was detected at the earliest time points (2 and 4 h), in the case of NaCl100 trehalose was detected only at the subsequent time points (6 and 8 h, respectively) whereas no trehalose was detected for the PEG100 treatments. This may lead to hypothesize that both stress agents inhibit the accumulation of trehalose during the first hours of imbibition (either by inhibiting its synthesis or by promoting its degradation), resulting in different profiles. Indeed, although the effect of NaCl is overcome at the later hours, the effect of PEG is not, at least not during the first 8 h of imbibition hereby investigated. Considering that the germination speed (T_50_) is also slowed-down during stress treatment (**Supplementary Figure 2D**) it is possible that the trehalose metabolism may be resumed later on during the germination process.

### *Medicago Truncatula Trehalose-6-Phosphate Synthase*, *Trehalose-6-Phosphatase*, and *Trehalase* Genes Are Differentially Upregulated in Response to Polyethylene Glycol and Sodium Chloride Treatments

**Figure 5:**
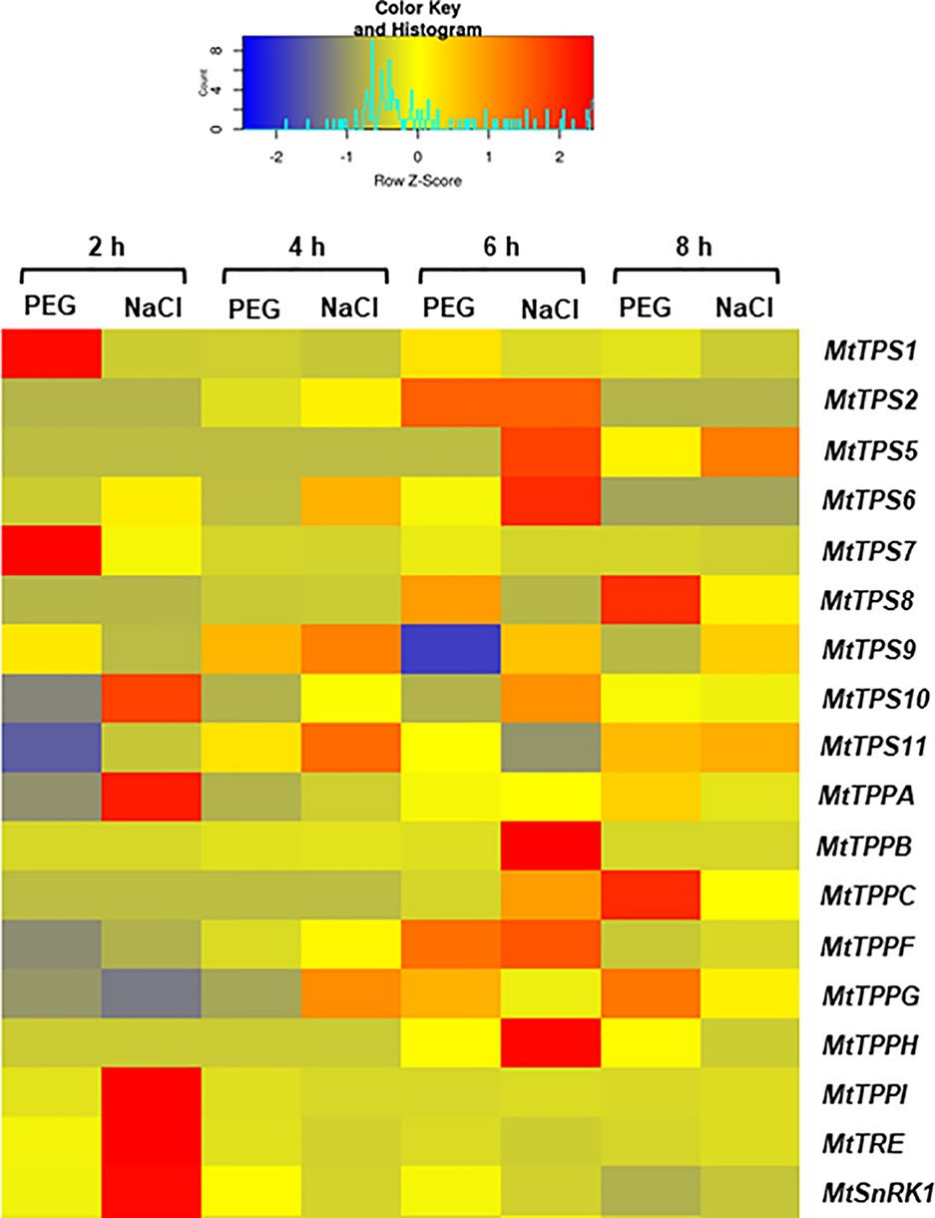
Heatmap representing the expression profiles of *Medicago truncatula MtTPS*, *MtTPP*, *MtTRE*, and *MtSnRK1* genes during seed imbibition carried out in the presence of polyethylene glycol and sodium chloride. The profiles are presented as fold-change to their respective controls (imbibition with water at each corresponding timepoint). Expression levels are represented by with color codes, where red is the highest expression while blue is the lowest expression.

Because trehalose levels had decreased during the treatment with PEG and NaCl, the expression patters of *MtTPS*, *MtTPP*, *MtTRE*, and *MtSnRK1* genes were evaluated also in response to these treatments during the first 8 h of imbibition. The data is represented as FC to control (**Figure 5**), where the control represents each corresponding time points of imbibition with water. Hence, it is possible to observe how the different isoforms are differentially upregulated in response to PEG and NaCl already at 2 h of imbibition. For instance, *MtTPS1* and *MtTPS7* were upregulated by PEG whereas *MtTPS10*, *MtTPPA*, *MtTPPI*, *MtTRE*, and *MtSnRK1* were upregulated by the NaCl treatment. While at 4 h of imbibition the expression patterns are more similar between PEG and NaCl treatments, after 6 h the situation seems considerably changed. In this case, there are two genes with a similar pattern of expression between the two treatments (*MtTPS2* and *MtTPPF*) whereas more genes are still upregulated in response to NaCl (*MtTPS5*, *MtTPS6*, *MtTPPB*, and *MtTPPH*) and one gene (*MtTPS9*) is downregulated under PEG treatment. Conversely, after 8 h of imbibition, the PEG treatment seems to affect more genes (*e.g.*, upregulation of *MtTPS8*, *MtTPPC*) than the NaCl treatment. Similar to what observed during seed imbibition with water (**Figure 2**), a preferential expression of *TPS* and *TPP* isoforms is maintained also under stress conditions. Except *MtTPS2* and *MtTPPF* (upregulated by both PEG and NaCl at 6 h), most differentially expressed isoforms do not overlap during exposure to PEG and NaCl. This indicates that different isoforms can be specifically modulated by the two stress agents; e.g., *MtTPS1*, *MtTPS7*, *MtTPS8*, *MtTPPPC* are upregulated by PEG treatments while *MtTPS5*, *MtTPS6*, *MtTPS10*, *MtTPPA*, *MtTPPB*, *MtTPPH*, *MtTPPI* are upregulated by NaCl treatments. In addition, it is possible to evidence a small set of genes having a potential impact as precocious hallmarks of the seed response to stress during imbibition. Indeed, when using gene expression as a tool to predict seed quality and reduce screening costs, it is mandatory to have informative (quality-related) profiles within the shortest timeframe soon after the beginning of imbibition. Among the tested genes, only *MtTPS1* and *MtTPS7* showed upregulation exclusively at 2 h of imbibition in response to the osmotic agent PEG while in the case of salt stress the *MtTPS10*, *MtTPPA*, *MtTPPI*, and *MtTRE*, genes were upregulated at 2 h. This set of genes deserve further investigation to better assess their role as indicators of seed vigor or stress responses during the early phases of seed germination. It is worth noting that both PEG and NaCl are routinely used as priming agents (although at considerably lower concentration as the ones used in our study) by seed technologists. The genes hereby identified to be specifically responsive to these chemicals during seed rehydration might be used as sensors to discriminate between the different levels of stress imposed by osmo- or halo-priming, hence choosing concentrations which are not deleterious to the germination process.

### Integrative Data Analysis Reveals Correlations Between Gene Expression Profiles and Trehalose Content

**Figure 6:**
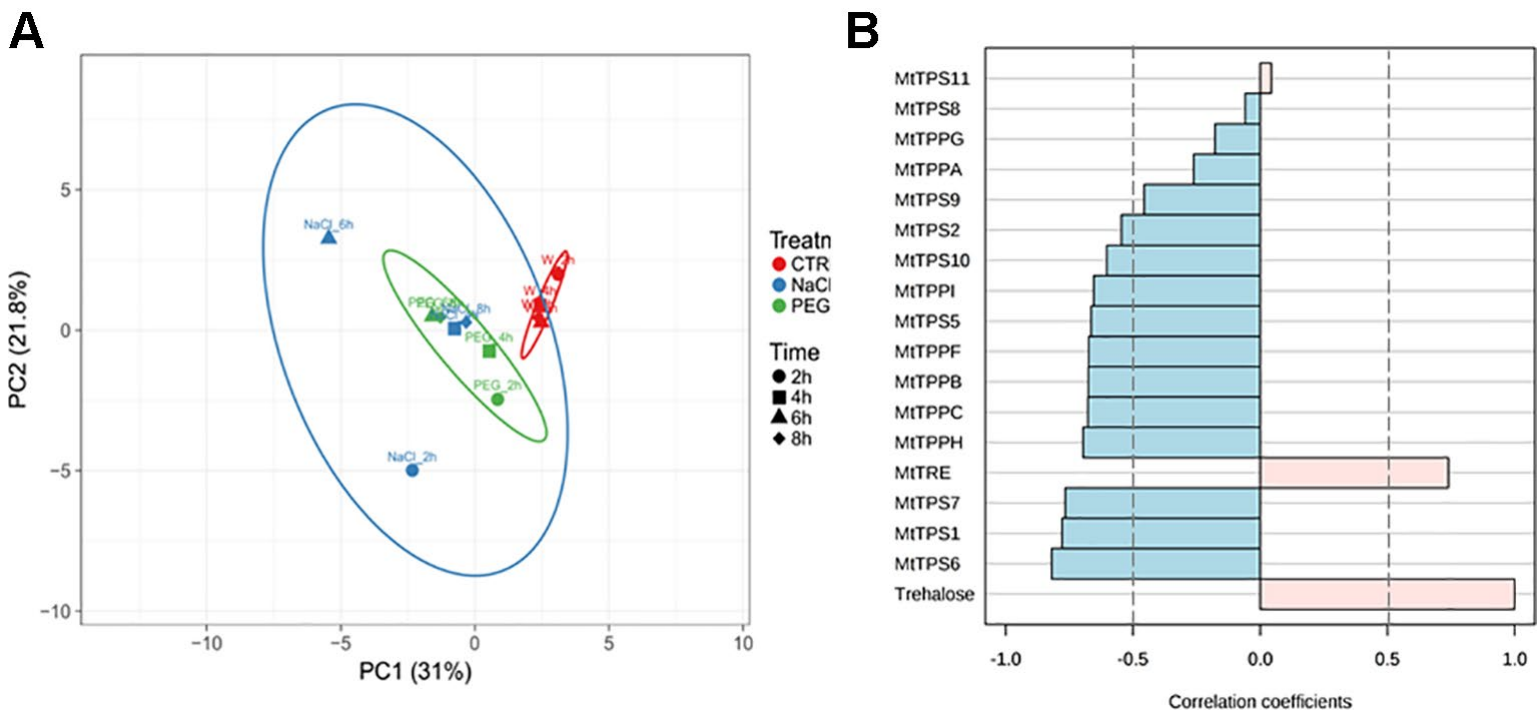
Integrative data analysis. **(A)** Principal component analysis loading plot explaining the distribution of samples based on the imposed conditions and measured variables. **(B)** Pattern search distribution of samples using all the data collected from gene expression profiles and trehalose detection at the indicated treatments and time points.

To better understand how samples behave comparatively, a PCA analysis was performed taking into consideration the imposed treatments and imbibition timepoints (as distinctive parameters) along with all measured variables (the levels of ROS, trehalose, and the expression profiles of all tested gene). The two main principal components extracted accounted for 52.8% of the variance (**Figure 6A**). Unit variance scaling is applied to rows; singular value decomposition (SVD) with imputation is used to calculate principal components. X and Y axis show principal component 1 (PC1) and principal component 2 (PC2) that explain 21.8 and 30% of the total variance, respectively. Prediction ellipses are such that with probability 0.95, a new observation from the same group will fall inside the ellipse. Based on how the samples are clustered, it seems that the effect of the imposed stresses is bigger that the effect of the time points, except for NaCl where some timepoints (4 and 8 h) are clustered together with PEG.

Additionally, we wanted to analyze which genes had the most significant correlation to the “trehalose” variable. A pattern search analysis was performed using all the data collected from gene expression profiles and trehalose detection at the indicated treatments and timepoints. Significant negative correlations are assumed when the correlation coefficient ≤ −0.5 whereas significant positive correlations are assumed when the correlation coefficient ≥ 0.5. This analysis showed that while most of the TPS and TPP isoforms (except for TPS8, TPS9, TPS11, TPPA, TPPG) were negatively correlated with the trehalose content, only TRE was positively correlated with this variable (**Figure 6B**). This pattern follows the regular flow of the metabolic reactions where a low activity of TPS and TPP would result in reduced levels of trehalose accumulation whereas enhanced TRE activity would result in trehalose degradation. Certainly, these correlations should be treated cautiously as it is difficult to hypothesize a direct connection between gene expression and metabolite levels without measuring the enzymatic activities/protein levels and other intermediates.

## Discussion

The present work provides a snapshot of the trehalose pathway focusing on the seed pre-germinative metabolism in the legume *M. truncatula*. Firstly, a bioinformatic analysis was carried out to retrieve the genes of the trehalose biosynthetic pathway and nine *MtTPS*s, eight *MtTPP*s, and one *MtTRE* were identified (**Figure 1**). The qRT-PCR analysis carried in *M. truncatula* seeds subjected to imbibition with water revealed a temporal distribution and preferential expression of specific *MtTPS* and *MtTPP* isoforms throughout imbibition (**Figure 2**), as seen in *Arabidopsis* as well (**Supplementary Figure 1**). A comparison between *M. truncatula* and *Arabidopsis* in terms of expression of the *TPS* and *TPP* gene families throughout seed imbibition revealed a variegated picture with predominant species-specific distinctive profiles. Only a few common features were noticed, as in the case of *TPS8* and *TPPF* genes that were highly expressed in both *M. truncatula* and *Arabidopsis* seeds within the first 4 h of imbibition. This may indicate that certain isoforms are more required than others during seed imbibition and that these may have a contribution to carbohydrate reallocation during metabolic reactivation. For instance, high levels of sucrose had been correlated with enhanced TPS activity and inhibition of SnRK1 (through T6P) which lead to the allocation of resources to promote active growth. Conversely, low levels of sucrose promote TPP and SnRK1 activity with the consequent resource allocations from source to sink (Smeekens, 2015). During *M. truncatula* seed imbibition in the presence of water, several metabolite changes were observed in the carbohydrate metabolic pathway, as seen in **Figure 3**. Namely, the increase in the levels of some sugars (*e.g.*, sucrose, UDP-glucose, mannose, ribulose, maltose) during seed imbibition points at the need for enhanced energy supply during this crucial phase of germination. Moreover, the increasing accumulation of glucuronate and the reduction of gluconate may reflect a diversion of glucuronate to the phosphoinositide pathway. This pathway was shown to be more active during seed imbibition, where, through the PI3K activity, the NADPH oxidase is activated, subsequently leading to the high generation of ROS (Liu et al., 2012), as highlighted also in our analyses (**Figure 4A**). Other indications come from a recent metabolomic study on mung bean (*Vigna radiata*) seed germination and sprouting showing that sugars strongly contribute to the high energy supply needed for sprout development and further metabolites synthesis (Chen et al., 2019). On the other hand, the role of trehalose as source of energy is quite elusive. It is known that the flux through the trehalose pathway is more than a 1.000-fold lower than the sucrose pathway where uridine diphosphate glucose (UDP-glucose) is released from sucrose to support main aspects of growth, including cell wall biosynthesis (Paul et al., 2018).

The trehalose content hereby detected in the *M. truncatula* seeds and quantified by HPLC is in agreement with a previous report describing the sugar profiles in wheat seeds and seedlings (El-Bashiti et al., 2005). The decrease in trehalose content suggests that *M. truncatula* seeds (both control and treated samples) are utilizing the sugar within the first two hours of imbibition. It is possible that trehalose needs to be removed for other reasons related to its role in desiccated seeds. Indeed, Singer and Lindquist (1998) reported that trehalose not only stabilizes proteins in yeast cells exposed to heat shock but also avoids the aggregation of denatured proteins. The sugar maintains proteins in a partially-folded state that can be reversed by molecular chaperones. In this case, the presence of trehalose interferes with protein refolding and the sugar must be rapidly hydrolyzed when physiological conditions are restored. Exposure to NaCl or PEG caused trehalose to disappear at 2 h of imbibition, suggesting for the need of TRE activity in this short temporal frame; however, in terms of gene expression profiles, upregulation of *MtTRE* was observed only in the case of NaCl treatments. The peculiar case of PEG is difficult to explain, although the possibility that the *MtTRE* gene could be upregulated even earlier than 2 h should be subsequently verified. Such a response in a short time further suggests for the role of trehalose as an energy source rather than osmoprotectant. Indeed, Queiroz and Cazetta (2016) showed that in maize seeds germinating under water restriction, trehalose was utilized instead of being accumulated. According to these authors, the sugar was preferentially used in energy metabolism for supporting germination and synthesis of proline in the embryo axis. Besides, the recent advancements in the field of trehalose metabolism establish a link with some intriguing physiological aspects, such as the regulation of source-sink communications at the whole plant level in response to stress to ensure the proper distribution of carbon nutrients in the organs (Rodrigues et al., 2019). From an applicational point of view, the source-sink optimization can be easily related to increased crop yield and resilience (Paul et al., 2017). Specifically, in the case of seed germination, it has been demonstrated that rice OsTPP7 modulates the sucrose levels during germination under anoxic stress by regulating the source (endosperm reserve) to sink (embryo axis–coleoptile growth) resource allocation, and thus promoting early seed vigor (Kretzschmar et al., 2015).

In the attempt to link the metabolite profile with the gene expression patterns, it should be noticed that five genes were up-regulated at 2 h of imbibition in the NaCl-treated seeds, among which *MtTRE*. The possibility that up-regulation of *MtTRE* gene took place even earlier during imbibition cannot be ruled out and this might explain the sudden drop in trehalose observed in the imbibed seed compared to dry seed. On the other hand, it seems that the response of *M. truncatula* seeds exposed to NaCl was much more complex in terms of trehalose metabolism since other players, distinct from *MtTRE*, were clearly involved. Thus, the observed up-regulation of *MtTPS10*, *MtTPPA*, and *MtTTPI* genes in response to salt stress might reflect, in a more general way, the trehalose turnover in the context of the sugar metabolic pathways ruled by *MtSnRK1*. The *MtSnRK1* expression is inhibited upon imbibition with water and PEG, but with salt stress it is highly expressed at 2 h after imbibition. This may reflect that salt is inducing a higher control on the seed metabolism. This may also explain the high expression of some of the genes of the trehalose pathway 6 h after imbibition under salt stress, in a way to modulate (through T6P) the activity of SnRK1. The different pattern of expression found under stress conditions, in particular under NaCl, for *MtTPS*s and *MtTPP*s (**Figure 4**) is well-corroborated by PCA (**Figure 6A**). The picture emerging from the analysis of PEG-treated seeds is more difficult to clarify since it seems that PEG may exert a matrix effect, so the concentration of trehalose in the seed samples treated with PEG could be underestimated. In this case no up-regulation of *MtTRE* gene occurred at 2 h of imbibition and, as previously speculated for the NaCl treatment, it is possible that the *MtTRE* gene expression would have been anticipated. It is also worth noting that the enzyme TRE is susceptible to post-translational modifications (*e.g.*, phosphorylation) that can increase the activity within minutes as reported in yeast cells exposed to osmotic stress (Fernández et al., 1997). Studies carried on the yeast enzyme suggest that phosphorylation of TRE *in vivo* is not sufficient for its activation, and that some proteins of the highly conserved 14-3-3 family bind to the phosphorylated residues in the N terminus of TRE, further stimulating its activity (Schepers et al., 2012). Whether this regulatory mechanism is used also by plants, it is still unknown. Studies on *M. truncatula* nodules have shown that the *MtTRE* expression is induced by the presence of the symbiotic bacteria under physiological conditions while salinity stress resulted in downregulation of the gene and subsequent accumulation of the metabolite (López et al., 2008).

To our knowledge, no data concerning the activity of TPS and TPP enzymes are available, not only in the context of seed imbibition but more generally *in planta*. The activity of the *Arabidopsis* TPS and TPP proteins was first tested upon heterologous expression in yeast (Ramon et al., 2009) and *E. coli* (Vandesteene et al., 2012) whereas *in planta* studies carried with overexpressing/knockout *Arabidopsis* lines were mostly focused on phenotype, trehalose, and T6P levels, rather than enzyme activity (Gaff, 1996; Avonce et al., 2004; Satoh-Nagasawa et al., 2006; Van Houtte et al., 2013). Zentella et al. (1999) also reported that TPS from the resurrection plant *Selaginella lepidophylla* was able to complement growth and stress-tolerance defects in a yeast *tps1* mutant. The concomitant induction of different enzymes of the trehalose biosynthetic pathway in response to stress has been reported in fungi, highlighting the relevance of trehalose mobilization. In *Schizosaccharomyces pombe*, increased tps1 or tpp1 expression and activity paralleled the enhanced trehalose levels following exposure to 0.75 M NaCl (Cansado et al., 1998; Franco et al., 2000). In a different report, Zähringer et al. (2000) showed that in *S. cerevisiae* cells treated with 0.5 M NaCl no significant changes in the trehalose content occurred, despite the increased enzyme activities of tps1 and nth1 (Neutral TRE). Other studies on the phytopathogenic fungus *Botrytis cinerea* (Döhlemann et al., 2006) and arbuscular mycorrhizal fungi (Ocón et al., 2007) showed lack of activation of trehalose metabolism in response to osmotic stress induced by NaCl.

This investigation also highlighted novel putative indicators of seed vigor or stress response. Such molecular hallmarks are genes whose expression is triggered during early imbibition, allowing the short-time prediction of seed quality, and required to support the design of tailored priming protocols by seed companies (Paparella et al., 2015). Looking at the expression profiles in response to NaCl treatment, only the *MtTPPA*, *MtTPPI*, and *MtTRE* genes seemed to address this feature, since enhanced transcript accumulation was observed at 2 h of imbibition. Similarly, the *MtTPS1* and *MtTPS7* genes were up-regulated at 2 h of imbibition in response to PEG. These genes appear as the most promising candidates for future strategies aimed at improving seed quality, although extensive validation on different species/seed lots will be needed.

Overall, the current study discloses some novel and original aspects dealing with the contribution of trehalose pathway in the context of the seed pre-germinative metabolism. A link was established between the seed ability to withstand oxidative stress generated by toxic concentrations of salt and osmotic agents and concomitant changes in the expression profiles of several trehalose-related genes. Among these genes, the class II TPS (e.g., *TPS7*, *TPS8* upregulated by PEG treatments and *TPS5*, *TPS6*, *TPS10*, upregulated by NaCl) represent the most promising candidates for future breeding strategies, worthy of future assessment for their involvement in the pre-germinative metabolism and, thus, their potential as tools to improve seed quality.

## Data Availability Statement

All datasets generated for this study are included in the article/**Supplementary Material**.

## Author Contributions

AM, AB, SA, and PF conceptualized the study, analyzed the data, and wrote the manuscript. AP and MC performed the bioinformatics, qRT-PCR, and ROS detection analyses. LG and DD performed the HPLC analyses. All authors read and approved the manuscript.

## Funding

This work was supported by Regione Lombardia D.G. Attività Produttive Ricerca e Innovazione-Struttura Asse 1 POR FSE 2007-2013, Project ID 4344853, CARIPLO Foundation (Action 3, Code 2013-1727) “Advanced Priming Technologies for the Lombardy Agro-Seed Industry-PRIMTECH”, and WAKE-APT project (Code 2016‐0723; “Seed Wake-up with Aptamers: A New Technology for Dormancy Release and Improved Seed Priming”). ITQB-UNL acknowledges funding from Fundação para a Ciência e Tecnologia, throughout the research unit GREEN-it “Bioresources for Sustainability” (UID/Multi/ 04551/2019) and S.S.A. PhD holder (DL57) contract.

## Conflict of Interest

The authors declare that the research was conducted in the absence of any commercial or financial relationships that could be construed as a potential conflict of interest.
