# Supplementary material for: A Snapshot of the Trehalose Pathway During Seed Imbibition in Medicago truncatula Reveals Temporal- and Stress-Dependent Shifts in Gene Expression Patterns Associated With Metabolite Changes
Source: Front Plant Sci. 2019 Dec 18;10:1590. doi: 10.3389/fpls.2019.01590 (PMC6930686; doi:10.3389/fpls.2019.01590)
Supplement: Supplementary file 1 [file Presentation_1.pdf]

Supplementary Material

A Snapshot of the Trehalose Pathway during Seed Imbibition in *Medicago truncatula* Reveals Temporal- and Stress-Dependent Shifts in Gene Expression Patterns Associated with Metabolite Changes

Anca Macovei[1,†], Andrea Pagano[1,†,‡], Michela Cappuccio[1,2], Lucia Gallotti[3], Daniele Dondi[3], Susana de Sousa Araújo[2], Pedro Salema Fevereiro[2], and Alma Balestrazzi[1,*]

[1]Country Department of Biology and Biotechnology 'L. Spallanzani', University of Pavia, Pavia, Italy

[2]Instituto De Tecnologia Quìmica e Biologica ITQB-UNL António Xavier (ITQB-UNL), Oeiras, Portugal

[3]Department of Chemistry, University of Pavia, Pavia, Italy

*** Correspondence:**
Alma Balestrazzi
alma.balestrazzi@unipv.it

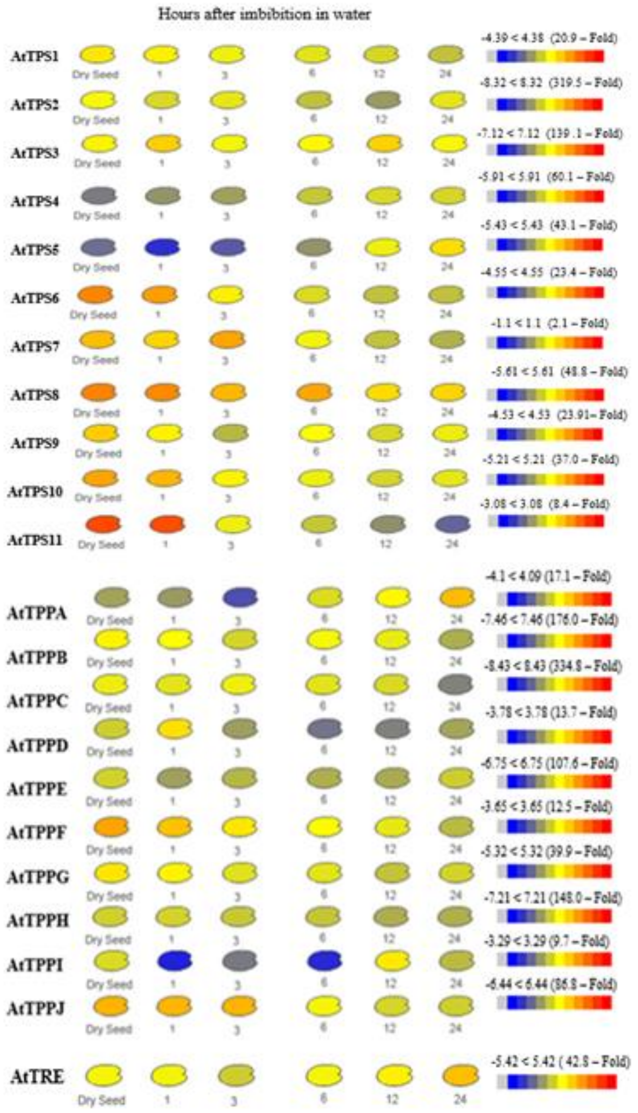

Supplementary Figure 1. Expression profiles of *A. thaliana AtTPS*, *AtTPP*, and *AtTRE* genes. Data is mined from AtGeneExpress microarray dataset and represented as pictographic image generated by Seed eFP Browser tool. Expression levels are represented by a color scale. The maximum expression level is indicated by the signal threshold above the color scale. The expression profiles of these genes were evaluated using the Seed eFP Browser tool (Winter et al., 2007; Bassel et al., 2008). In dry seeds, the most expressed isoforms are *AtTPS11*, *AtTPS8* and *AtTPS6*. The same profile is observed at 1 h of imbibition, adding a reduced expression of *AtTPS5*. At 3 h, the most expressed isoforms are *AtTPS7* and *AtTPS8*. At 6 h, *AtTPS8* shows the highest transcript level. As for the *TPP* gene family, *AtTPPF* and *AtTPPJ* are more expressed in dry seed while a relatively high expression is still visible at 1 h, 3 h and 6 h of imbibition. The expression level of *AtTPPI* appears reduced at 1 h and 6 h after imbibition with water. The *AtTRE* gene is slightly enhanced only after 24 h of imbibition.

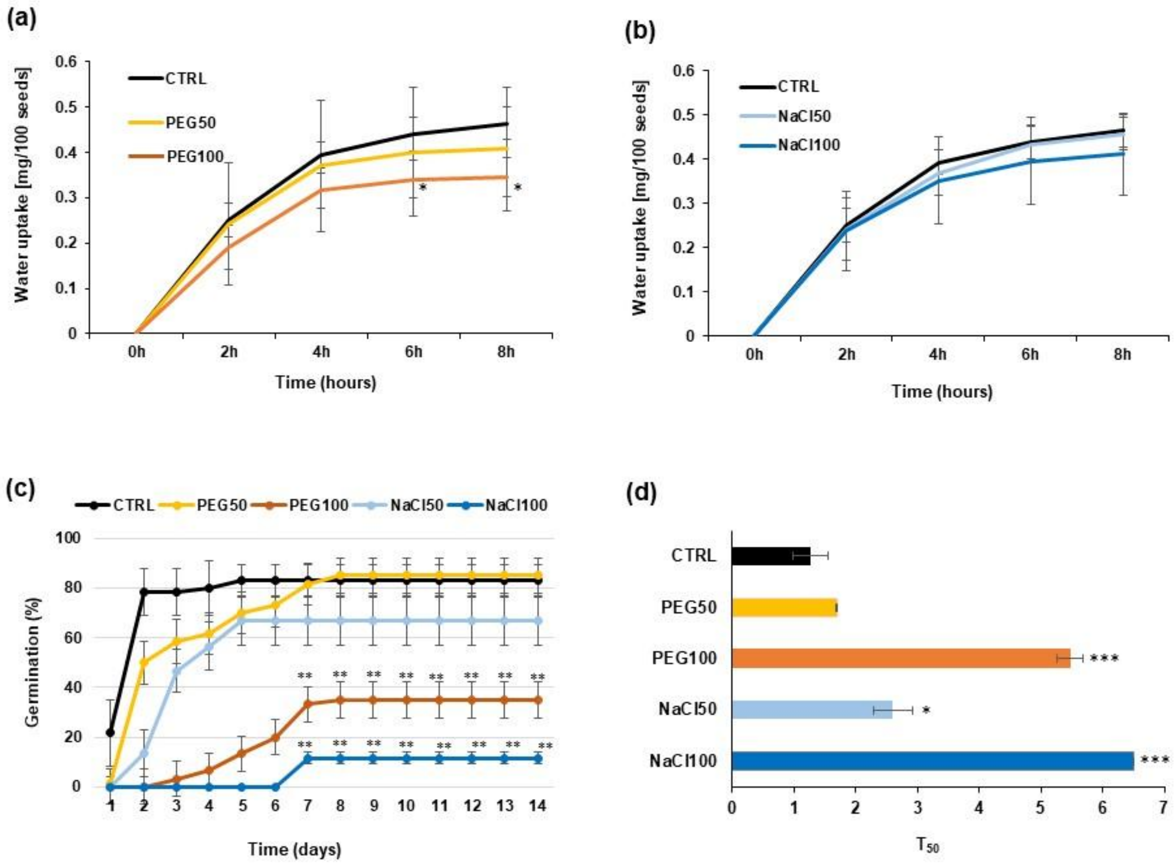

Supplementary Figure 2. Profiles of *M. truncatula* seed imbibition and germination under physiological and stress conditions. **(a)** Measurement of water uptake in control- (CTRL, water imbibition) and PEG-treated seeds. **(b)** Measurement of water uptake in control- and NaCl-treated seeds. **(c)** Percentage of seed germination. **(d)** The time necessary to reach 50% of germinated seeds (T_{50}). Significant differences between treatments (PEG, NaCl) and control (water) were assessed based on the Student *t*-test (‘*’ $p < 0.1$; ‘**’ $p < 0.05$; ‘***’ $p < 0.001$).

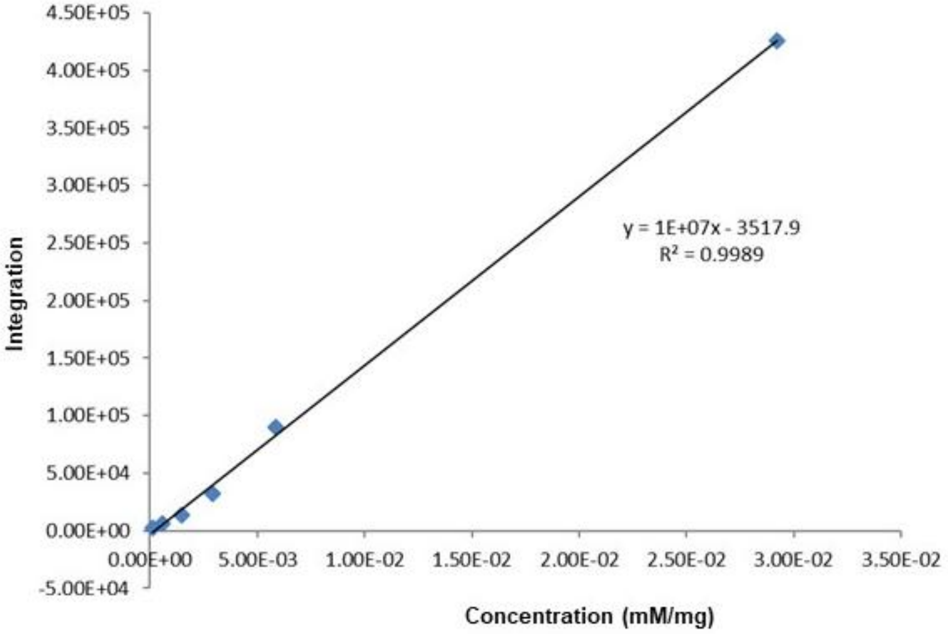

Supplementary Figure 3. Calibration curve used for the HPLC detection of trehalose content. The peaks of HPLC spectra were integrated and compared to the acquired trehalose standard signal. The concentration values were obtained on an average of three injections for each sample. The calibration curve was obtained for a concentration of trehalose in the range $3x10^{-2}$ – $1.5x10^{-4}$ M (R^2=0.998).

Supplementary Table 1. Oligonucleotide sequences used for the qRT-PCR reactions. For each primer pair, the PCR efficiency is given.

Gene	Forward Primer (5’→3’)	Reverse Primer (5’→3’)	Efficiency
MtACT	TCAATGTGCCTGCCATGTATGT	ACTCACACCGTCACCAGAATCC	1.70
MtTPS1	GACTGCTCCACATCTCTGC	GTCCGCACCTCACTGATCG	1.75
MtTPS2	CCAACAGGTCGTGATAGAACA	AGAAGGTAGCGGGCACTT	1.85
MtTPS5	TGAAGGCACAAAGGAAAGAGGA	CAGGCGACAGAGGAAGCAT	1.76
MtTPS6	TACCCCACCCAATCAAAATACA	AGAGAAGAAACAGCAACCAAAC	1.75
MtTPS7	GGTGGTCTCCATCGCTAAGTG	CATGCCGCAACTGCTTCT	1.69
MtTPS8	AGGAGTGATGGTTGGTATGTTGAGA	CCGAGGGGAGTTGGAAGGAA	1.83
MtTPS9	TCAGGAAGCAAAGAAAGAAGCA	CGAAACGAGGAACAGAGCGG	1.82
MtTPS10	TAGGGGACGAGGGAAAGACG	GGCAACAACATAGTAAGCAACT	1.83
MtTPS11	TGCTGTGAGGGATGGGATGA	GCTGAGTGAAGGCGAACAA	1.78
MtTPPA	ACGGAACTCTATTACGCTGGT	CTTCTTGCCCTGCTTGTCTG	1.73
MtTPPB	GTAGAAGGGGTCAAGGGGT	CTCCACCAATCGCCGCAA	1.83
MtTPPC	ACTCTTTTCCATCCTTCTGCGTT	GTCCTTCAATGTCACTCTCATCTT	1.88
MtTPPF	ATGAGGTCCGTAAGTTGCT	GCGTTGAGCCACCAAATCC	1.84
MtTPPG	TCATCTTCCGCTCCACCG	AGTTCCATCATAGTCCAAAAGC	1.83
MtTPPH	TCCCAACAAGATTCACCGC	GCCTTCATAGCATCCAACCA	1.78
MtTPPI	TGCCAGAGTTGAGAACCAC	CCTTGCCCTTGTCCCATTTA	1.64
MtTRE	GGAGATAATAGGACTGCTGAACAC	TGGTATTGCTGAGCCAGGAAT	1.78
MtSnRK1	GGCGTCTTCTTCAATCATCGTCA	GCATTCACATTTCCACCACCAG	1.81

Supplementary Table 2. List of *A. thaliana* and *M. truncatula* TPS (trehalose-6-phosphate synthase), TPP (trehalose-6-phosphate phosphatase) and TRE (trehalase) accessions. The percentage of similarity between *A. thaliana* and *M. truncatula* protein sequences is provided. Only the *M. truncatula* proteins with a similarity ≥ 50% with their *A. thaliana* orthologs were included. The orthologs presented complete protein domains have been selected for further analysis, hereby represented in bold.

**Name**	**A. *thaliana* accession**	***M. truncatula* accession**	**Similarity (%)**
TPS1	AT1G78580	**Medtr2g073260**	75.78
TPS2	AT1g16980	Medtr8g087910	68.09
		Medtr0034s0170	64.87
		Medtr2g073260	64.77
		**Medtr8g087930**	60.04
TPS3	AT1G17000	Medtr2g073260	61.08
		Medtr0034s0170	62.16
TPS4	AT4G27550	Medtr2g073260	63.18
		Medtr0034s0170	63.18
TPS5	AT4G17770	**Medtr1g109620**	77.40
		Medtr8g105740	72.16
		Medtr1g032730	71.31
		Medtr8g063790	64.08
		Medtr4g080160	63.21
		Medtr4g129270	56.63
		Medtr3g078210	52.25
		Medtr3g467030	50.43
TPS6	AT1G68020	**Medtr8g105740**	81.99
		Medtr1g032730	80.73
		Medtr1g109620	73.33
		Medtr8g063790	64.85
		Medtr4g080160	64.68
		Medtr4g129270	57.60
		Medtr3g078210	52.99
TPS7	AT1G06410	**Medtr4g080160**	77.65
		Medtr4g129270	56.14
TPS8	AT1G70290	Medtr8g063790	73.24
		Medtr8g105740	63.89
		Medtr1g032730	62.59

		Medtr1g109620	62.03
		Medtr4g080160	60.31
		Medtr4g129270	58.29
		**Medtr3g078210**	57.47
TPS9	AT1G23870	**Medtr8g063790**	75.32
		Medtr1g109620	63.85
		Medtr8g105740	63.74
		Medtr1g032730	63.40
		Medtr4g080160	59.48
		Medtr4g129270	59.04
TPS10	AT1G60140	Medtr8g063790	72.40
		Medtr8g105740	62.56
		**Medtr1g032730**	61.63
		Medtr1g109620	61.03
		Medtr4g080160	60.77
		Medtr4g129270	57.41
		Medtr3g078210	55.33
TPS11	AT2G18700	**Medtr4g129270**	63.40
		Medtr8g063790	60.12
		Medtr8g105740	59.16
		Medtr1g032730	58.06
		Medtr1g109620	57.75
		Medtr4g080160	56.41
		Medtr3g078210	51.93
TPPA	AT5G51460	**Medtr4g036090**	69.87
		Medtr3g008500	61.84
		Medtr4g101600	60.80
		Medtr4g036685	54.55
		Medtr8g090330	53.12
		Medtr3g074180	50.86
TPPB	AT1G78090	**Medtr3g074180**	62.99
		Medtr8g090330	62.57
		Medtr5g063080	60.93
		Medtr4g036090	51.54
		Medtr3g008500	50.56
TPPC	AT1G22210	Medtr8g090330	62.74
		Medtr3g074180	61.89
		**Medtr5g063080**	61.22

		Medtr3g008500	53.65
		Medtr4g036090	53.16
		Medtr4g101600	50.79
TPPD	AT1G35910	Medtr5g063080	60.78
		Medtr3g074180	60.23
		Medtr4g036090	55.81
		Medtr3g008500	55.21
		Medtr4g101600	55.11
TPPE	AT2G22190	Medtr8g090330	65.14
		Medtr5g063080	60.86
		Medtr3g074180	60.35
		Medtr4g101600	55.29
		Medtr4g036090	54.46
		Medtr3g008500	52.94
		**Medtr8g027765**	50.00
TPPF	AT4G12430	Medtr4g036090	65.47
		Medtr4g101600	64.64
		**Medtr3g008500**	64.29
		Medtr4g036090	63.81
		Medtr8g090330	54.17
		Medtr4g036685	52.40
		Medtr3g074180	51.34
		Medtr5g063080	51.32
		Medtr8g027765	50.98
TPPG	AT4G22590	Medtr4g036090	65.51
		Medtr4g101600	63.27
		Medtr3g008500	62.73
		Medtr8g090330	52.45
		**Medtr4g036685**	52.34
TPPH	AT4G39770	Medtr8g090330	68.60
		Medtr3g074180	61.61
		Medtr5g063080	60.93
		Medtr3g008500	56.50
		**Medtr4g101600**	55.59
		Medtr4g036090	55.09
TPPI	AT5G10100	**Medtr8g090330**	69.53
		Medtr3g074180	62.01

		Medtr5g063080<br>Medtr4g101600<br>Medtr4g036090<br>Medtr3g008500	58.24<br>52.23<br>51.27<br>50.00
TPPJ	AT5G65140	Medtr8g090330<br>Medtr3g074180<br>Medtr5g063080<br>Medtr4g101600	72.08<br>62.11<br>59.10<br>54.65
TRE	AT4G24040	**Medtr8g099985**	58.44
